# A novel glutaminase inhibitor-968 inhibits the migration and proliferation of non-small cell lung cancer cells by targeting EGFR/ERK signaling pathway

**DOI:** 10.18632/oncotarget.14188

**Published:** 2016-12-26

**Authors:** Tianyu Han, Meng Guo, Tingting Zhang, Mingxi Gan, Caifeng Xie, Jian-Bin Wang

**Affiliations:** ^1^ Institute of Translational Medicine, Nanchang University, Nanchang City, Jiangxi 330031, China; ^2^ School of Life Sciences, Nanchang University, Nanchang City, Jiangxi 330031, China; ^3^ Research Institute of Applied Biology, Shanxi University, Taiyuan, Shanxi 030006, China

**Keywords:** glutamine metabolism, glutaminase inhibitor, epidermal growth factor receptor, autophagy, non-small cell lung cancer

## Abstract

Metabolic reprogramming is critical for cancer cell proliferation. Glutaminolysis which provides cancer cells with bioenergetics and intermediates for macromolecular synthesis have been intensively studied in recent years. Glutaminase C (GAC) is the first and rate-limiting enzyme in glutaminolysis and plays important roles in cancer initiation and progression. We previously screened a small molecule named 968, a specific inhibitor of GAC, to block the proliferation of human breast cancer cells. In this study, we found that 968 effectively inhibited NSCLC cell proliferation and migration and arrested G0/G1 phase of cell cycle. Furthermore, we demonstrated that 968 inhibited the EGFR/ERK pathway via decreasing the expression of EGFR and phospho-ERK. Apart from this, we discovered that 968 treatment induced autophagy to protect cells against apoptosis and the combination of 968 with autophagy inhibitor Chloroquine (CQ) had synergistic effects on the growth of NSCLC cells. Thus, our study pointed out a new therapeutic strategy for NSCLC treatment by combination of 968 with CQ.

## INTRODUCTION

Cancer cells always consume a large amount of glucose at a high rate and produce most of the glucose-derived carbon as lactate when compared with normal cells even with adequate oxygen, a phenomenon termed the aerobic glycolysis or “Warburg effect” [1]. Several decades later after “Warburg effect” discovered, another metabolic pathway known as glutaminolysis was identified as an important cancer-related metabolism [2, 3]. Glutamine, the most abundant amino acid in the plasma has been demonstrated as an indispensable non-essential amino acid in cancer cells. It provides bioenergetics and intermediates for macromolecular synthesis through entering into tricarboxylic acid (TCA) cycle and thus is crucial for supporting uncontrolled cell proliferation, cell migration and invasion into distant tissue [4].

As the first enzyme in glutaminolysis, glutaminase also acts as the rate-limiting enzyme. There are two isoforms of glutaminase known as liver-type glutaminase (LGA) and kidney-type glutaminase (KGA) [5, 6]. In kidney-type glutaminase, a spliced variant known as glutaminase C (GAC) has been demonstrated to be closely related to cancer initiation and progression [7]. In human B lymphoma cells and prostate cancer cells, glutamine deprivation affected tumor cell growth more severely than glucose withdrawal and GAC knockdown significantly inhibited cell proliferation [8]. In breast cancer cells, GAC knockdown affected cancer cell growth under low serum condition and inhibited colony formation in soft agar [9]. In non-small cell lung cancer (NSCLC), loss of GAC affected NSCLC cell growth more than loss of KGA, and this effect was more apparent in glutamine dependent cells than glutamine independent cells [10]. GAC has also been demonstrated as a biomarker for pathologic diagnosis and prognosis of hepatocellular carcinoma [11]. All these results highlight the crucial role of GAC in cancer cells and the potential of targeting GAC as a new therapeutic strategy to cancer treatment.

Lung cancer is now the leading cause of deaths related to malignant tumours in China [12]. The two main classification of lung cancer depended upon tumour histology are small cell lung carcinoma (SCLC) and non-small cell lung carcinoma (NSCLC) [13]. SCLC patients usually respond better to radiation and chemotherapy while NSCLC patients are less responsive. So a new and effective therapeutic strategy is urgent for NSCLC treatment.

In our previous study, we showed that a novel small chemical molecular 5-(3-bromo-4-(dimethylamino)phenyl)-2,2-dimethyl-2,3,5,6-tetrahydrobenzo[a]phenanthridin-4(1H)-one, designated as 968, could block Rho GTPase-dependent cellular transformation and the growth of human breast cancer cells and B lymphoma cells without affecting normal cells by inhibiting GAC activity [9]. However, the precise mechanism by which altered glutamine metabolism resulted in an antitumor effect is still not fully elucidated. In this study, we reported that the novel glutaminase inhibitor-968 effectively inhibited NSCLC cells growth and migration and induced G1-phase cell cycle arrests. We elucidated that the inhibition effects of 968 on cancer cell growth was regulated by EGFR/ERK signaling pathway. Except for this, we also found that 968 treatment induced autophagy by up-regulating Beclin1 and more significantly, 968 combined with Chloroquine treatment had a more powerful inhibition effects on cancer cell growth than treated with 968 alone. We next demonstrated that these effects were due to the enhanced inhibition on glutaminase activity. Taken together, blocking GAC activity may be an effective way to treat NSCLC and inhibiting 968 induced autophagy may enhance the therapeutic effects of 968.

## RESULTS

### Glutaminase inhibitor-968 effectively inhibits the growth and migration of NSCLC cells

**Figure 1:**
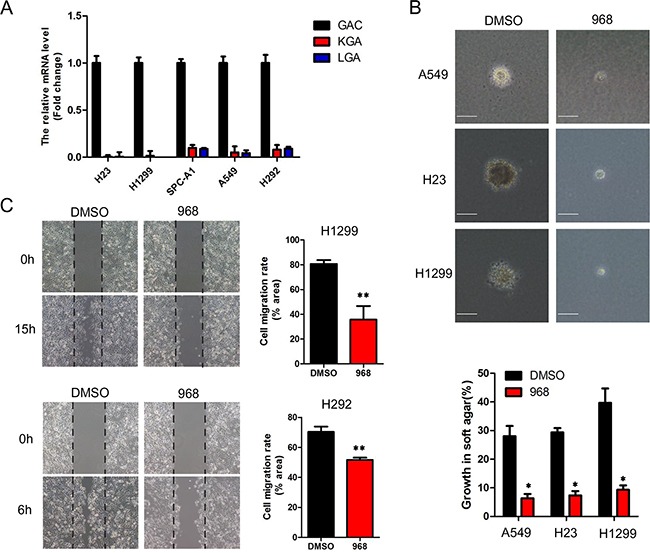
968 effectively inhibits the growth and migration of NSCLC cells **A**. The total RNA of NSCLC cells were extracted and reverse transcribed to cDNA. The mRNA levels of GAC, KGA and LGA were quantified by real-time RT-PCR using 2^ΔΔCT^ method. Data represent the average of three independent experiments (mean±SD). **B**. Anchorage-independent growth of A549, H23 and H1299 cells treated with 10 μM of 968 was evaluated by the colony formation efficiency (CFE). Colonies larger than 50μm were counted. CFE is determined by formula as: (number of colonies formed/number of cells incubated) ×100%. Scale bar indicates 100 μM. Data represent the average of three independent experiments (mean±SD). *, p<0.05 vs. DMSO. **C**. The cell migration of H1299 and H292 treated with 10 μM of 968 were evaluated by wound healing assay. Cell migration rate was quantified by measuring the difference in area between the leading edge at the initiation of the experiment. The wound area was assessed by the ImageJ software. Data represent the average of three independent experiments (mean±SD). **, p<0.01 vs. DMSO.

There are two isoforms of glutaminase in human cells: kidney type glutaminase, which is known as GLS1, and liver type glutaminase which is known as GLS2. There are also two spliced variants of GLS1, known as KGA and GAC. We first detected the expression pattern of glutaminase in non-small cell lung cancer cells. As can be seen in Figure 1A, the expression of GAC is much higher than the other isoforms of glutaminase. GAC has been demonstrated to be the predominant isoform of glutaminase in breast cancer cells, and our previous studies showed that the GAC specific inhibitor 968 effectively inhibited the growth of breast cancer cells. So we examined the effects of 968 on NSCLC cells. As shown in Supplementary Figure 1A-1F, the viability of NSCLC cells (A549, Spc-A1, H23, H292, H1299) were effectively inhibited when the concentrations of 968 were≥10μM (p<0.05). However, there were only slightly inhibitory effects on the viability of human bronchial epithelial cells (HBE), even the concentration of 968 reached 40μM. Then, we assessed the effects of 968 on the anchorage-independent growth of NSCLC cells. A549, H23 and H1299 treated with 968 formed fewer and smaller colonies in soft agar than the cells treated with DMSO (Figure 1B). To explore possible effects of 968 on NSCLC cell migration, wound healing assay was performed and showed that the migration rate was markedly attenuated when H292 or H1299 cells were treated by 10μM 968 (Figure 1C).

### 968 treatment induced G_1_/G_0_-phase cell cycle arrest

**Figure 2:**
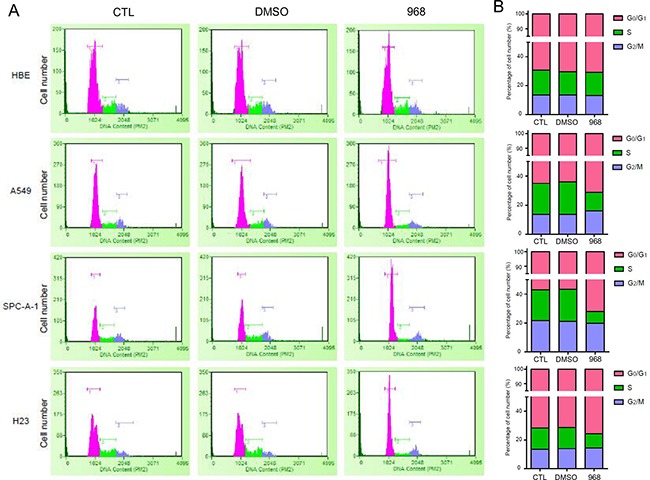
968 treatment induced G1/G0-phase cell cycle arrest **A**. HBE, A549, Spc-A1, H23 were treated with 10 μM 968 or DMSO for 48h. Cells were collected and analyzed by flow cytometry after propidium iodide staining. **B**. The quantification of cell number in each phase of the cell cycle were derived from Figure 2A and marked with different colors (pink: G_1_/G_0_ phase, green: S phase, and blue: G_2_/M phase).

In order to identify the cause of the cell growth inhibition treated with 968, cell cycle was analyzed by flow cytometry after cytometry propidium iodide staining. HBE, A549, Spc-A1, H23 were treated with 968 for 48 hours. Then, cell cycle phase distribution was examined. The cells in G_0_/G_1_ phase were remarkably increased in A549, Spc-A1 and H23 treated with 968. However, there was no significant changes in the cell cycle phases of HBE cells when treated with 968 (Figure 2A). These results were more obvious when the cell numbers of each cell cycle phase were counted (Figure 2B). Cyclin-dependent kinases (CDKs) and their activators, Cyclins, drive the cell cycle progression, we then checked the mRNA levels of the CDKs and Cyclins in SPC-A1 cells after treated with 968. As shown in Supplementary Figure 2A-2C, the genes CDK4, CDK6 and CCND2, which were associated with G0/G1 phase of cell cycle were increased in a concentration-dependent manner, meanwhile the cell cycle gene CCNE2, which drives the cell cycle progression from G1 phase to S phase was declined in dose-dependence. CCNA2 and CDC2, which were associated with G2/M phase were also reduced. These results demonstrated that the growth inhibition caused by 968 treatment might be due to the G_0_/G_1_ cell cycle arrest for NSCLC cells. To confirm that the cell cycle arrest was caused by inhibition of glutamine metabolism, we knocked down GAC using specific siRNAs in NSCLC cells and examined the cell cycle phase distribution. The cells in G_0_/G_1_ phase were remarkably increased in A549 and H23 cells when GAC was knocked down. However, no significant changes were seen in the cell cycle phases of HBE cells when knocking down GAC (Supplementary Figure 3A, 3B).

### 968 inhibited NSCLC cell growth through down-regulating EGFR/ERK pathway

**Figure 3:**
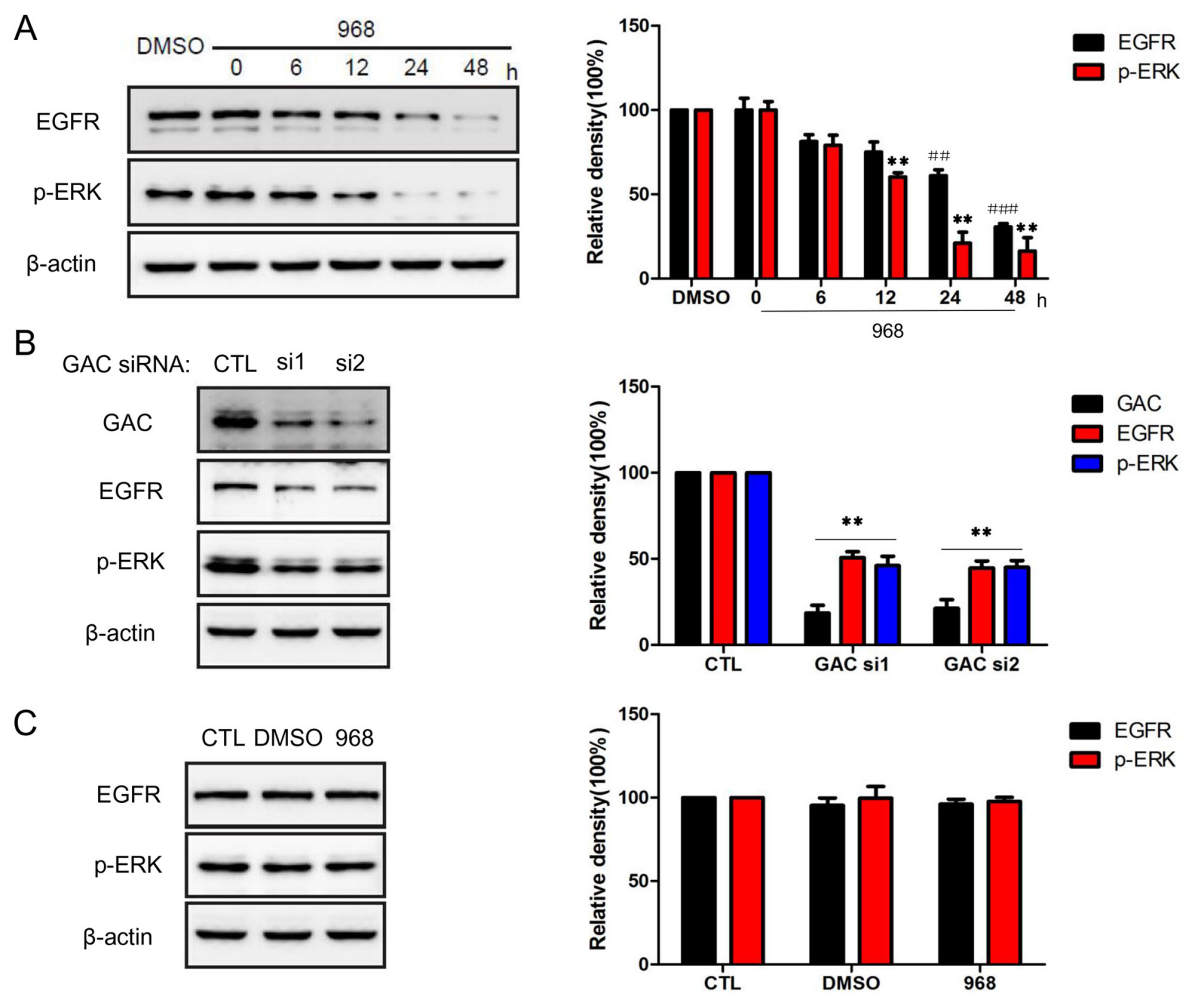
968 treatment down-regulated EGFR/ERK pathway **A**. H23 cells were treated with 10 μM 968 for indicated times and examined by immunoblot. Quantification of the data from Western blot was expressed as the mean ± SD from three independent experiments. p-ERK: **, p<0.01 vs. DMSO. EGFR: ##, p<0.01 vs. DMSO; ###, p<0.001 vs. DMSO. **B**. EGFR and phospho-ERK protein level of H23 cells with GAC knocking down were examined by immunoblot. Quantification of the data from Western blot was expressed as the mean ± SD from three independent experiments. **, p<0.01 vs. Control siRNA. **C**. The impacts of 968 on the expression of EGFR and phospho-ERK in HBE cells were determined by immunoblot. Quantification of the data from Western blot was expressed as the mean ± SD from three independent experiments.

EGFR/ERK signaling is an important pathway in the regulation of cellular proliferation, and plays an indispensable role in tumor initiation and development. In order to investigate the molecular mechanism of the growth inhibition effects of 968, we examined the influence of 968 treatment on EGFR/ERK pathway. As shown in Figure 3A, the expression levels of both EGFR and phospho-ERK1/2 were greatly reduced by 968 treatment in a time-dependent manner. Furthermore, the expression level of EGFR and phospho-ERK1/2 was declined significantly when knocking-down GAC by specific siRNAs (Figure 3B). However, there were no effects on the expression of EGFR and phospho-ERK1/2 in HBE cells (Figure 3C). To further explore the mechanism by which 968 decreased the EGFR expression, we detected the effects of 968 treatment on the mRNA level of EGFR in H23 cells. Supplementary Figure 4A showed that the mRNA level of EGFR did not changed significantly when treated with 968. This indicated that 968 might decrease EGFR expression through promoting the protein degradation. We next examined the ubiquitination level of EGFR. A significantly increased ubiquitination of EGFR was observed when H23 cells were treated with 968 (Supplementary Figure 4B). However, 968 treatment had no effects on the ubiquitination of EGFR in HBE cells (Supplementary Figure 4C). So we concluded that 968 inhibited NSCLC cell growth through down-regulating EGFR/ERK pathway which is an important pathway in tumorigenesis.

### 968 treatment induced autophagy by up-regulating beclin1 in NSCLC cells

**Figure 4:**
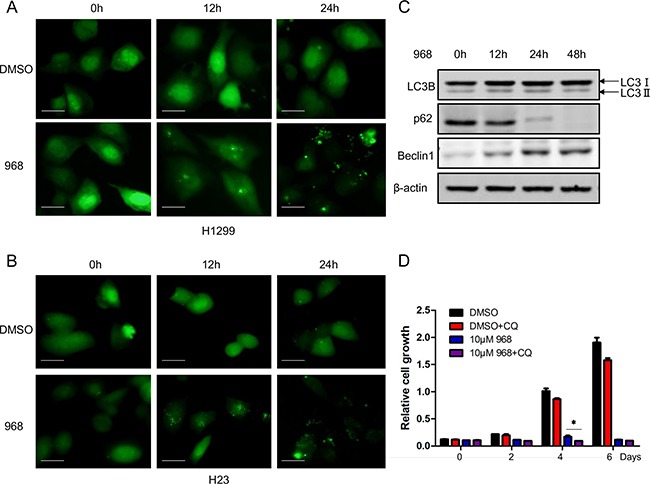
968 treatment induced autophagy in NSCLC cells **A, B**. H1299 and H23 were transfected with GFP tagged LC3 expression plasmid. 24h later, the cells were treated with 10μM 968 or DMSO for different time. The distribution of punctate LC3 was observed using immunofluorescence microscopy. Scale bar indicates 20 μM. **C**. H1299 cells transfected with GFP tagged LC3 were treated with 10μM 968 for different time and total cell lysates were collected and performed immunoblot using different antibodies. **D**. H1299 cells were treated with 10μM 968 alone or combined treatment with 10μM 968 and 20μM Chloroquine (CQ) for different time and cell growth assay was used to assess the growth rate. Data represent the average of three independent experiments (mean±SD). *, p<0.05 vs. 10μM 968.

Autophagy is an important cellular process in which damaged organelles, cytosolic proteins are degraded within lysosomes. In tumor development, autophagy has been demonstrated as a critical pathway and thus it has been recognized as an attractive therapeutic target. We next examined whether 968 had any effects on autophagy. H1299, H23 cells expressing GFP-LC3B were treated with 968 for different time. The GFP-LC3B puncta were significantly induced after 968 treatment compared with DMSO treatment (Figure 4A, 4B). The expression level of LC3-IIwas also increased in a time dependent manner accompanied by decreased p62 expression level in H1299 cells (Figure 4C). Beclin1 is a critical autophagy regulator that affects the formation of autophagosomes in mammalian cells. We found that 968 treatment could increased the expression level of beclin1 and thus promoted autophagy progression (Figure 4C). As autophagy plays a dual role in cancer progression, we next want to figure out the role of autophagy induced by 968 treatment. Chloroquine (CQ) can suppress lysosomal degradation by increasing lysosomal pH and thus be used as an autophagy inhibitor. As shown in Figure 4D, although 968 treated alone apparently suppressed the proliferation of H1299 cells, a more significantly inhibitory effect was observed when the cells were treated with 968 combined with 20μM CQ. These results indicated that the autophagy induced by 968 treatment could relieve the toxicity of 968 on NSCLC cells.

### Autophagy inhibition promotes 968 induced cell death

**Figure 5:**
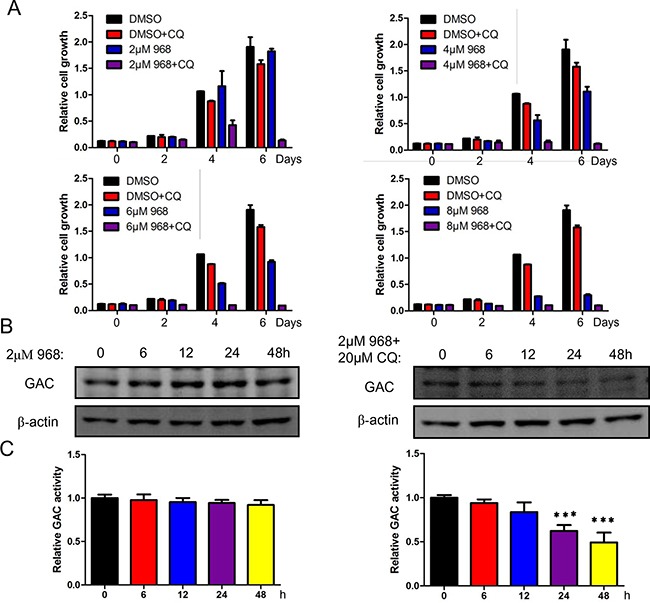
Autophagy inhibition promotes 968 induced cell death **A**. H1299 cells were treated with different concentrations of 968 combined with 20μM CQ for different time and cell growth assay was used to examine the growth rate. Data represent the average of three independent experiments (mean±SD). **B**. H1299 cells were treated with 2μM 968 alone or combined treatment with 2μM 968 and 20μM Chloroquine (CQ) for different time and total cell lysates were collected and performed immunoblot using different antibodies. **C**. H1299 cells were treated with 2μM 968 alone or combined treatment with 2μM 968 and 20μM Chloroquine (CQ) for different time. After that, mitochondria were isolated and glutaminase activity was performed. Data represent the average of three independent experiments (mean±SD). ***, p<0.001.

As autophagy inhibition promoted 968 induced cell death, we next treated H1299 cells with various concentrations of 968 alone or combined with 20μM CQ. As can be seen in Figure 5A, low dose of 968 treated alone had only slightly inhibitory effects on cell growth, however, when combined treatment with CQ, the cell growth was remarkably inhibited, even at 2μM 968 treatment. To define the mechanism of this effect, we examined the expression of GAC. 2μM 968 treatment had only slight effects on GAC expression while 2μM 968 plus 20μM CQ treatment significantly decreased the expression of GAC even treated for only 12 hours (Figure 5B). We next tested the activity of GAC and found that 2μM 968 plus 20μM CQ treatment remarkably reduced GAC activity when treated for 24 hours, however, 2μM 968 treatment alone had no effects on GAC activity even treated for 48 hours (Figure 5C). These results provided a new therapeutic strategy that inhibiting glutaminase activity combined with autophagy suppression might be a promising strategy towards curing cancer.

## DISCUSSION

As the most important compensatory pathway of “Warburg effect”, glutamine metabolism has received great research interest. Recent studies discovered new roles of glutamine that beyond its roles as metabolic substrate. *Nicklin et al*. reported that activation of mTORC1 required bidirectional transport of glutamine: glutamine was imported into the cells by SCL1A5 and then exported out of the cells by SCL7A5 which was accompanied by importing essential amino acids and subsequent activation of mTOR pathway [14]. Apart from this, glutamine can influence gene expression through regulating the expression of transcription factors like c-Myc and c-Jun [15, 16]. So glutamine metabolism is far more complicated than we previously thought, and this may be one of the reasons why glutamine metabolism became so popular in recent years.

As the importance of altered glutamine metabolism has been recognized, great efforts were made to exploit this metabolic change for cancer treatment. The glutamine analogues L-DON (6-diazo-5-oxo-l-norleucine) was able to compete with glutamine in glutaminolysis and had received promising results in preclinical studies. However this agent also inhibited the metabolic pathways that utilizing glutamine as a substrate, so the tumor inhibition effects needed careful evaluation [17]. Recently, two allosteric inhibitors of glutaminase have been reported, BPTES [bis-2-(5-phenylacetamido-1,2,4-thiadiazol-2-yl) ethyl sulfide] and compound 968 [9, 18]. Structural studies indicated that BPTES (and BPTES analogs) affected formation of an active glutaminase tetramer through the binding of two inhibitor molecules at a set of loops at the interface of the homodimer [19]. However, the moderate potency, poor metabolic stability and low solubility of BPTES limit its potential for clinical development. Previously, we found a new allosteric inhibitor of GAC, compound 968, which blocked breast cancer cell proliferation and the growth of tumors in mouse xenograft models but little effects were observed on normal cells [9]. Computational docking studies suggested this dibenzophenanthridine series of glutaminase inhibitors might bind to an interface between the two monomers rather than the glutamine binding pocket, but experimental evidence for this was still lacking [20]. In this study, we showed that 968 inhibited the growth and blocked the migration of NSCLC cells, which only slightly affected the normal cells. These results suggest that the novel allosteric inhibitor of GAC will be the most promising compound for treating NSCLC.

The precise mechanism by which altered glutamine metabolism resulted in an antitumor effect is still not fully elucidated. It was reported that inhibition of glutamine metabolism prevented Dlx-2-, TGF-β, Wnt-, and Snail-induced EMT and glycolytic switch in MCF-7 cells [21]. Another study reported that compound 968 could alter the expression of cancer-related genes through affecting the gene-specific histone H4K16ac and H3K4me3 patterns in MDA-MB-231 cancer cells [22]. In this study, we found that the inhibitory effects of 968 treatment was through EGFR/ERK pathway. EGFR activation promotes tumor cell proliferation, angiogenesis, invasion, and metastasis [23]. The activation of EGFR leads to the downstream phosphorylation of ERK in lung cancer cells [24]. Herein, 968 decreased expression of EGFR and reduced the phosphorylation of ERK1/2. This result was confirmed by inhibiting glutamine metabolism through down-regulating GAC expression using GAC specific siRNAs. We further demonstrated that 968 could increase the ubiquitination level of EGFR in NSCLC cells, thus promoting the protein degradation of EGFR. So we concluded that glutamine metabolism inhibition blocked the EGFR/ERK1/2 signaling pathway and thus inhibited NSCLC cell growth, migration.

Autophagy is a highly conserved degradation process that cytoplasmic contents are encapsulated by autophagosomes and degraded in lysosomes, thus it is important to maintain cellular homeostasis under stress conditions [25, 26]. Recently, autophagy was demonstrated as a critical pathway in cancer initiation and progression and also an important factor for cancer cells exposured to anticancer drugs acquiring drug-resistance ability. Until now, there is still a conflict of opinions towards the roles of autophagy in cancer development [27]. In NSCLC cells, the preclinical studies demonstrated that the combination of hydroxychloroquine (HCQ) and erlotinib showed no advantage over erlotinib alone in reducing tumor growth [28]. Here we found that 968 treatment could induce autophagy in NSCLC cells. Chloroquine (CQ) treatment enhanced the inhibition effect of 968 treatment, indicating that autophagy induction by 968 treatment was used to protect cells against apoptosis. We also found that combined treatment with 968 and CQ inhibited cell growth more significantly than 968 treated alone and low dose of 968 treatment could have an obvious inhibition effect on cell growth when combined with CQ, however, there were only slight inhibition effects on cell growth under low dose of 968 treatment. The molecular mechanism of the combined treatment was the enhanced inhibition of GAC activity. These findings highlight a new therapeutic strategy that targeting glutamine metabolism and autophagy may be a promising way towards cancer treatment.

## MATERIALS AND METHODS

### Reagents

Compound 968 was purchased from Calbiochem (Merck Millipore, Darmstadt, Germany). Chloroquine (CQ) was bought from Sigma (Sigma, C7698). Mouse monoclonal β-actin antibody was purchased from Proteintech (66009-1-lg). Mouse monoclonal EGFR antibody was obtained from BD Biosciences (610017). Commercial rabbit polyclonal antibodies anti-LC3B and anti-ubiqutin were purchased from Proteintech (18725-1-AP, 10201-2-AP). SQSTM1/p62 and Beclin1 mouse monoclonal antibodies were ordered from OriGene (TA502127, TA502127). Rabbit polyclonal GAC antibody was purchased from Abcam (ab93434). Phospho-p44/42 MAPK (Thr202/Tyr204) monoclonal antibody (p-ERK) was ordered from Cell Signaling (9106). Crystal violet and other analytical grade chemicals were purchased from Sigma-Aldrich.

### DNA constructs

Human LC3B cDNA was isolated by PCR using the cDNA library from H1299 cells with the following primers: 5′-GCCTCGAGCATGCCGTCGGAGAAGACCTT-3′ (forward) and 5′-GCGGTACCTTA CACTGACAATTTCATCC-3′(reverse). The construct was cloned into pEGFP-C2 vector in XhoI and KpnI restriction sites and verified by sequencing.

### Cell culture

Human bronchial epithelial cells (HBE) were cultured in Airway epithelial cell basal medium supplemented with bronchial/tracheal epithelial cell growth kit (ATCC). The five types of human non-small cell lung cancer lines, H23, H1299, A549, H292, SPC-A1 were cultured in RPMI 1640 medium supplemented with 10% FBS (GIBICO), at 37°C with 5% CO_2_.

### MTT assay

The cell viability was detected by MTT assay. Cells were seeded in 96-well plate for 24 hours. Then the cells were treated with different concentrations of 968 in a total volume of 200μl per well for 48 hours. After that, 20μl MTT(5mg/ml) solution was added to each well and incubated for 4 hours at 37°C with 5% CO_2_. Then, discard the culture medium and add 150μl DMSO to each well and incubated on an end-over shaker for 20 minutes and measure the absorbance at 570nm. Measurements were done in triplicate.

### Soft agar assay

For soft agar assay, 10^4^ cells were mixed with RPMI 1640 supplemented with 10% FBS and 0.3% agarose and plated on top of a solidified layer of RPMI 1640 supplemented with 10% FBS and 0.5% agarose. 1ml of RPMI 1640 supplemented with 10% FBS and 0.3% agarose were added to the cells weekly and colonies larger than 50μm were counted.

### Cell migration assay

Cells were seeded in 6-well plate for 24 hours to form a monolayer. Then, a straight line was draw across the monolayer using a 200μl pipet tips and the well was washed three times by PBS, and the line was photographed using a microscope (Olympus, IX71) as the start of the time point. After that, RPMI 1640 with 1% FBS was added to the well. The photos were taken at the indicated time point.

### Cell proliferation assay

For cell proliferation assay with 968 treatment, cells were seeded in 24-well plates at 3000 cells per well in 0.5 ml medium with 10% FBS. On the following day, the medium was changed to RPMI 1640 with different treatment. The medium was changed every 2 days. At the indicated time points, cells were fixed in 3.7% formaldehyde and stained with 0.1% crystal violet. Dye was extracted with 10% acetic acid and the relative proliferation was determined by the absorbance at 595nm.

### RNA interference

The siRNAs targeting GLS1 (HSS104192, HSS104193, HSS178458) was purchased from Thermo Fisher (Cat.#1299001). HSS104192, HSS104193 were used for knocking down GAC. NSCLC cell lines were transiently transfected with the indicated siRNAs or Stealth RNAi™ siRNA Negative Control using Superfectin II *In Vitro* siRNA Transfection Reagent (Shanghai Pufei Biotech). The knockdown efficiency was determined by western blot using indicated antibodies.

### Mitochondrial isolation and glutaminase activity assay

The detailed procedures for this part were carried out as previously described [9]. Briefly, Mitochondial isolation was conducted using the mitochondria isolation kit from QIAGEN following the manufacturer's instructions. 2×10^7^ cells were collected and centrifuged at 500g for 10 min at 4°C. The cell pellets were suspended in 2ml of lysis buffer and incubated on ice for 10 min using an end-over-end shaker. The cell lysates were centrifuged at 1000×g for 10 min at 4°C, then the pellets were resuspended in 1.5 ml disruption buffer using a blunt-ended, 23-gauge needle and a syringe. The suspension was centrifuged at 6000×g for 20 min at 4°C. The pellets were resuspended in 100μl of storage buffer and assayed for glutaminase activity. Briefly, 20μl resuspended mitochondrial lysate were added in a reaction buffer I [57mM Tris-Acetate (pH8.6), 0.225 mM EDTA, 17mM glutamine] and incubated by rotating at 37°C for 1 hour. The reaction was stopped by adding 10μl ice-cold 3M hydrogen chloride (HCl) and incubated on ice for 5 minutes. Then, 10μl quenched reaction mixture was added to a reaction buffer II containing 114 mM Tris-HCl (pH9.4), 0.35 mM adenosine diphosphate (ADP), 1.7 mM nicotinamide adenine dinucleotide (NAD), 6.3 U/ml glutamate dehydrogenase, 1% hydrazine to give a final volume of 230μl and incubated at room temperature for 45 minutes. The formation of NADH was detected by the absorbance at 340 nm against water blank. Measurements were done in triplicate.

### Quantitative RT-PCR

Total RNA was extracted using TRIzol reagent (Invitrogen) and 1 μg total RNA was performed reverse transcription using PrimeScript RT reagent kit with gDNA eraser (TaKaRa), according to the manufacturer's instructions. Quantitative RT-PCR was performed with SYBR Green dye using (Applied Biosystems). The relative amount of cDNA was calculated by the comparative Ct method using GAPDH as a control. PCR reactions were performed in triplicate. The primers used in quantitative RT-PCR were listed in Supplementary Table 1.

### Western blot

Protein extracts were prepared using NP-40 lysis buffer containing phosphatase and protease inhibitors, the protein concentration of the cell lysates were determined by BCA protein assay kit. The cell lysates were then subjected to SDS-PAGE followed by immunoblot using indicated antibodies.

### Immunofluorescence

Cells transfected with GFP-LC3B were treated with 968 for different time point and observed under immunofluorescence microscopy.

### Cell cycle analyze

Cells were seeded in 6-well plate and cultured for 24 hours. Then the cells were treated with 968 or DMSO for 48 hours. After that, the cells were trypsinized, washed with ice-cold PBS for three times and fixed with 70% ethanol for 2 hours on ice. Then the cells were washed with PBS and resuspended in 1 ml of staining solution containing 20 μg/ml propidium iodide (PI), 200 μg/ml RNase A and 1% Triton X-100 in PBS. Samples were incubated for 15 minutes at 37°C and then analyzed by the Millipore Guava easyCyte™ flow cytometer (Millipore).

### Statistical analysis

Data are presented as means ± SD. One-way ANOVA and unpaired t-test were used to make the statistical comparisons, *P*-values≤0.05 were considered to be statistically significant.

## SUPPLEMENTARY MATERIALS FIGURES AND TABLES




