# Supplementary material for: A novel glutaminase inhibitor-968 inhibits the migration and proliferation of non-small cell lung cancer cells by targeting EGFR/ERK signaling pathway
Source: Oncotarget. 2016 Dec 26;8(17):28063–73. doi: 10.18632/oncotarget.14188 (PMC5438631; doi:10.18632/oncotarget.14188)
Supplement: Supplementary file 1 [file oncotarget-08-28063-s001.pdf]

A novel glutaminase inhibitor-968 inhibits the migration and proliferation of non-small cell lung cancer cells by targeting EGFR/ERK signaling pathway

SUPPLEMENTARY FIGURES AND TABLE

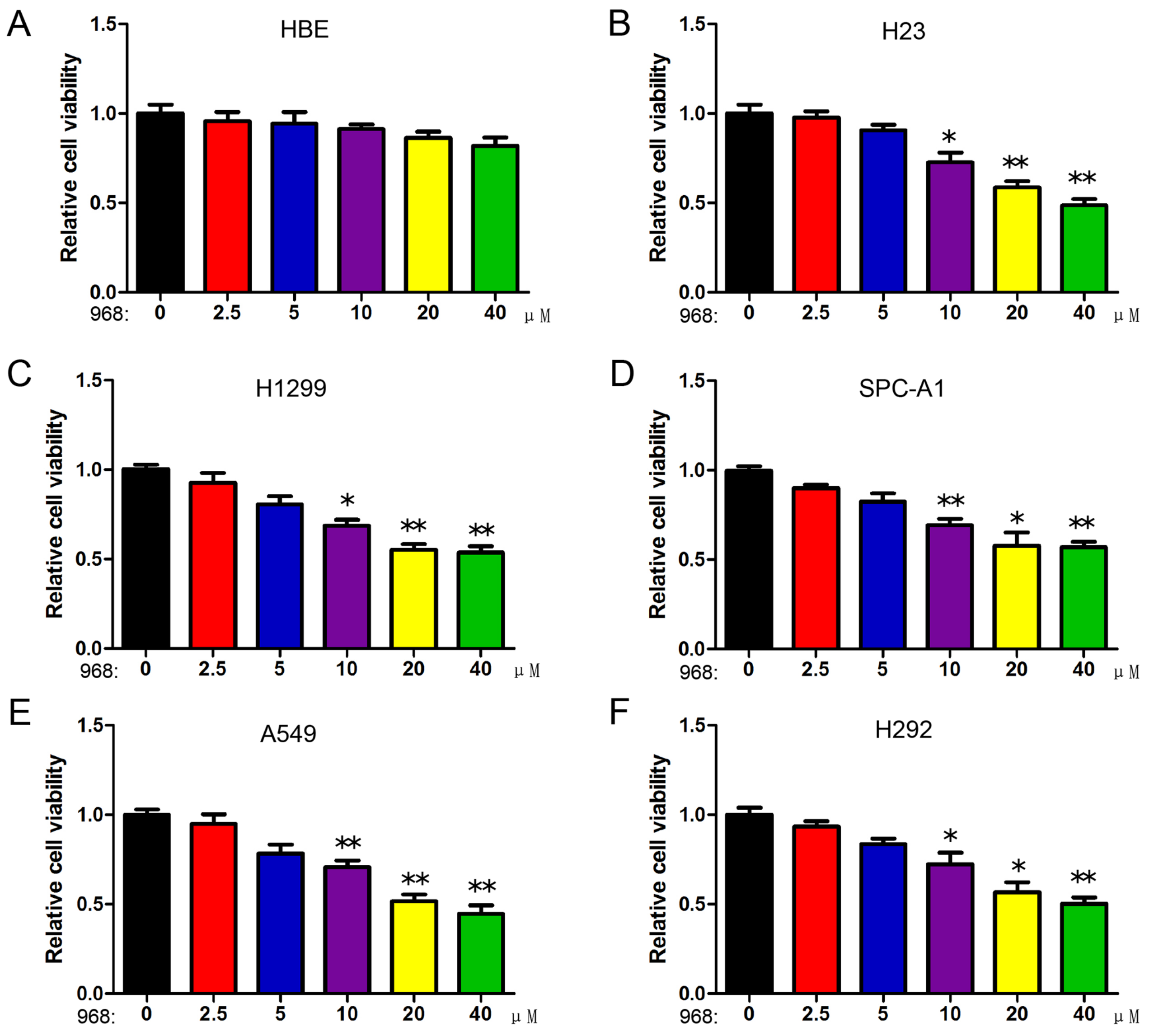

Supplementary Figure 1: 968 treatment affects the viability of NSCLC cells. A-F. HBE cells and NSCLC cells were treated with different concentrations of 968 for 2 days and the cell viability was determined using MTT assay. Data represent the average of three independent experiments (mean±SD). *, $p<0.05$ vs. DMSO; **, $p<0.01$ vs. DMSO.

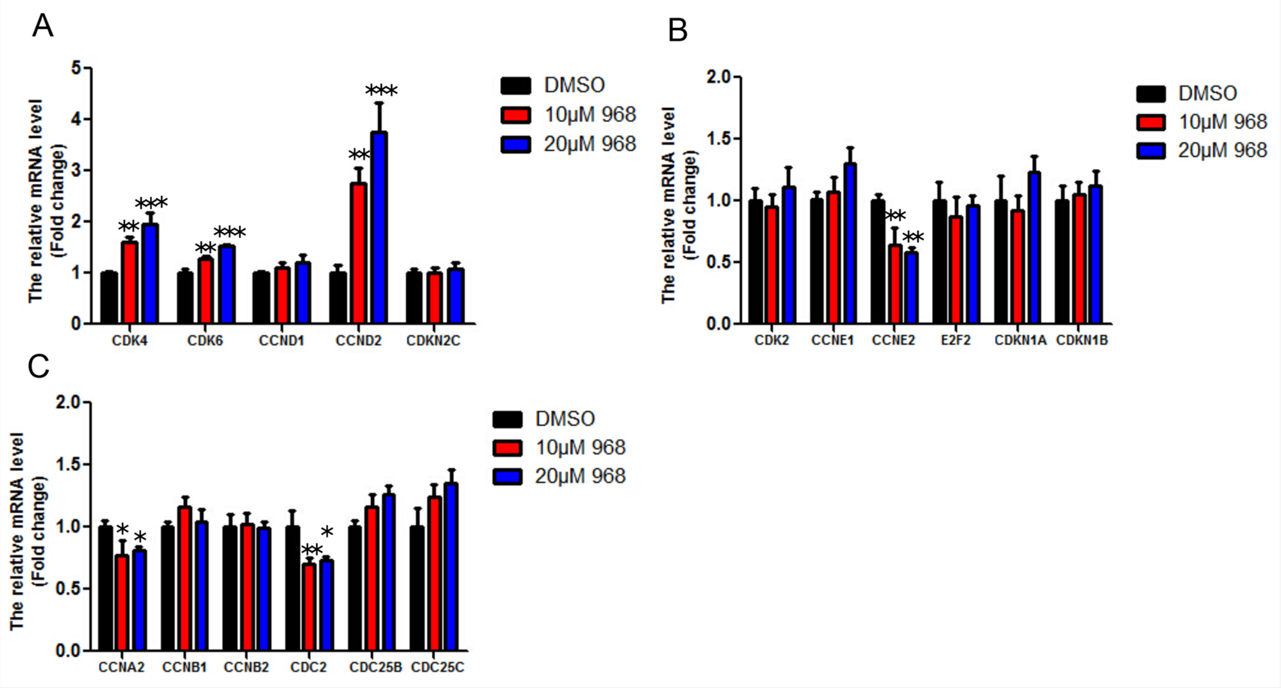

Supplementary Figure 2: 968 treatment affects cell cycle associated genes. A-C. The mRNA expression of cell cycle associated genes in SPC-A1 cells treated with different concentrations of 968 (10μM and 20μM) were tested by quantitative Real-Time PCR. Data represent the average of three independent experiments (mean±SD). *, $p<0.05$ vs. DMSO; **, $p<0.01$ vs. DMSO; ***, $p<0.001$ vs. DMSO.

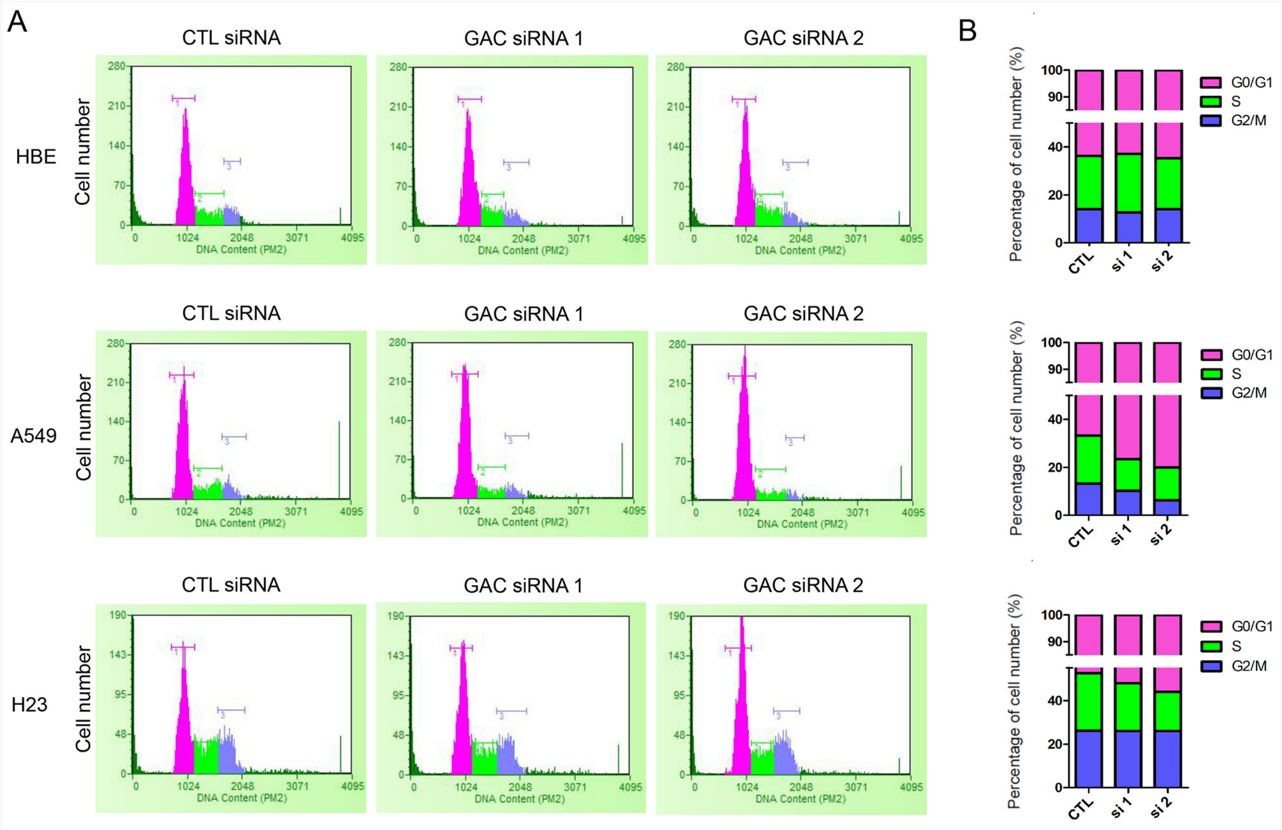

Supplementary Figure 3: GAC knockdown induced G1/G0-phase cell cycle arrest. A. HBE, A549 and H23 cells were transiently transfected with control siRNA or GAC siRNAs. 48 hours later, cells were collected and analyzed by flow cytometry after propidium iodide staining. **B.** The quantification of cell number in each phase of the cell cycle were derived from Supplementary Figure 3A and marked with different colors (pink: G_1/G_0 phase, green: S phase, and blue: G_2/M phase).

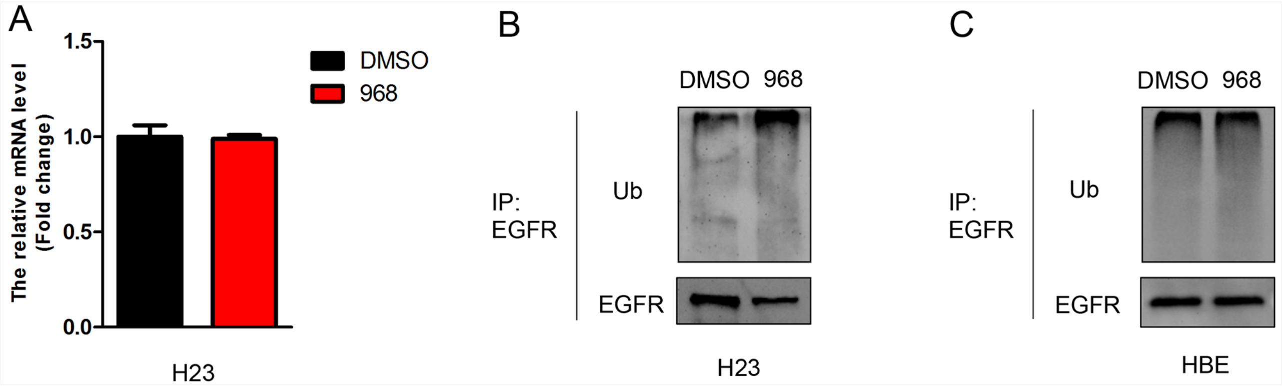

Supplementary Figure 4: 968 decreased EGFR expression through promoting the ubiquitin mediated degradation of EGFR. A. H23 cells were treated with 10μM 968 or DMSO for 48 hours. The mRNA expression of EGFR was tested by quantitative Real-Time PCR. Data represent the average of three independent experiments (mean±SD). **B, C.** H23 cells (B) and HBE cells (C) were transiently transfected with pcDNA3.0-EGFR plasmid. 24 hours later, the cells were treated with 10μM 968 or DMSO for 48 hours. Then, the cells were lysed and immunoprecipitation was performed using anti-EGFR antibody. The protein expression was examined by western blot using indicated antibodies.

Supplementary Table 1: The primers used in quantitative RT-PCR.

See Supplementary File 1
