# Supplementary material for: A novel glutaminase inhibitor-968 inhibits the migration and proliferation of non-small cell lung cancer cells by targeting EGFR/ERK signaling pathway
Source: Oncotarget. 2016 Dec 26;8(17):28063–73. doi: 10.18632/oncotarget.14188 (PMC5438631; doi:10.18632/oncotarget.14188)
Supplement: Supplementary file 2 [file oncotarget-08-28063-s002.doc]

**Primers used in quantitative RT-PCR:**

| **GAC** | Forward primer | AGGTGGTGATCAAAGGCATTC |
| --- | --- | --- |
|  | Reverse primer | GCTTTTCTCTCCCAGACTTTCC |
| **KGA** | Forward primer | TCCCCAAGGACAGGTGGAATA |
|  | Reverse primer | CCTTGAGGTGTGTACTGGACT |
| **LGA** | Forward primer | TCTCTTCCGAAAGTGTGTGAGC |
|  | Reverse primer | CCGTGAACTCCTCAAAATCAGG |
| **GAPDH** | Forward primer | ACAACTTTGGTATCGTGGAAGG |
|  | Reverse primer | GCCATCACGCCACAGTTTC |
| **CDK4** | Forward primer | ATGGCTACCTCTCGATATGAGC |
|  | Reverse primer | CATTGGGGACTCTCACACTCT |
| **CDK6** | Forward primer | GCTGACCAGCAGTACGAATG |
|  | Reverse primer | GCACACATCAAACAACCTGACC |
| **CCND1** | Forward primer | GCTGCGAAGTGGAAACCATC |
|  | Reverse primer | CCTCCTTCTGCACACATTTGAA |
| **CCND2** | Forward primer | ACCTTCCGCAGTGCTCCTA |
|  | Reverse primer | CCCAGCCAAGAAACGGTCC |
| **CDKN2C** | Forward primer | GGGGACCTAGAGCAACTTACT |
|  | Reverse primer | CAGCGCAGTCCTTCCAAAT |
| **CDK2** | Forward primer | CCAGGAGTTACTTCTATGCCTGA |
|  | Reverse primer | TTCATCCAGGGGAGGTACAAC |
| **CCNE1** | Forward primer | AAGGAGCGGGACACCATGA |
|  | Reverse primer | ACGGTCACGTTTGCCTTCC |
| **CCNE2** | Forward primer | TCAAGACGAAGTAGCCGTTTAC |
|  | Reverse primer | TGACATCCTGGGTAGTTTTCCTC |
| **E2F2** | Forward primer | CGTCCCTGAGTTCCCAACC |
|  | Reverse primer | GCGAAGTGTCATACCGAGTCTT |
| **CDKN1A** | Forward primer | TGTCCGTCAGAACCCATGC |
|  | Reverse primer | AAAGTCGAAGTTCCATCGCTC |
| **CDKN1B** | Forward primer | AACGTGCGAGTGTCTAACGG |
|  | Reverse primer | CCCTCTAGGGGTTTGTGATTCT |
| **CCNA2** | Forward primer | CGCTGGCGGTACTGAAGTC |
|  | Reverse primer | GAGGAACGGTGACATGCTCAT |
| **CCNB1** | Forward primer | AATAAGGCGAAGATCAACATGGC |
|  | Reverse primer | TTTGTTACCAATGTCCCCAAGAG |
| **CCNB2** | Forward primer | CCGACGGTGTCCAGTGATTT |
|  | Reverse primer | TGTTGTTTTGGTGGGTTGAACT |
| **CDC2** | Forward primer | AAACTACAGGTCAAGTGGTAGCC |
|  | Reverse primer | TCCTGCATAAGCACATCCTGA |
| **CDC25B** | Forward primer | ACGCACCTATCCCTGTCTC |
|  | Reverse primer | CTGGAAGCGTCTGATGGCAA |
| **CDC25C** | Forward primer | TCTACGGAACTCTTCTCATCCAC |
|  | Reverse primer | TCCAGGAGCAGGTTTAACATTTT |
